# Under-five mortality rate variation between the Health and Demographic Surveillance System (HDSS) and Demographic and Health Survey (DHS) approaches

**DOI:** 10.1186/s12889-016-3786-2

**Published:** 2016-10-24

**Authors:** Amare Deribew, John Ojal, Boniface Karia, Evasius Bauni, Mark Oteinde

**Affiliations:** 1 KEMRI-Wellcome Trust Research Programme, Kilifi, Kenya; 2 Nuffield Department of Clinical Medicine, University of Oxford, Oxford, UK; 3 INDEPTH, Accra, Ghana; 4 St. Paul Millennium Medical College, Addis Ababa, Ethiopia

## Abstract

**Background:**

Several low and middle-income countries (LMIC) use Demographic and Health Surveys (DHS) and/or Health and Demographic Surveillance System (HDSS) to monitor the health of their population. The level and trends of under-five mortality rates could be different in the HDSS sites compared to the DHS reports. In this study, we investigated the change in under-five mortality rates overtime in the HDSS sites and the corresponding DHS reports in eight countries and 13 sites.

**Methods:**

Under-five mortality rates in the HDSS sites were determined using number of under-five deaths (numerator) and live births (denominator). The trends and annualized rate of change (ARC) of under-five mortality rates in the HDSS sites and the DHS reports were compared by fitting exponential function.

**Results:**

Under-five mortality rates declined substantially in most of the sites during the last 10–15 years. Ten out of 13 (77 %) HDSS sites have consistently lower under-five mortality rates than the DHS under-five mortality rates. In the Kilifi HDSS in Kenya, under-five mortality rate declined by 65.6 % between 2003 and 2014 with ARC of 12.2 % (95 % CI: 9.4–15.0). In the same period, the DHS under-five mortality rate in the Coastal region of Kenya declined by 50.8 % with ARC of 6 % (95 % CI: 2.0–9.0). The under-five mortality rate reduction in the Mlomp (78.1 %) and Niakhar (80.8 %) HDSS sites in Senegal during 1993–2012 was significantly higher than the mortality decline observed in the DHS report during the same period. On the other hand, the Kisumu HDSS in Kenya had lower under-five mortality reduction (15.8 %) compared to the mortality reduction observed in the DHS report (27.7 %) during 2003–2008. Under-five mortality rate rose by 27 % in the Agincourt HDSS in South Africa between 1998 to 2003 that was contrary to the 18 % under-five mortality reduction in the DHS report during the same period.

**Conclusions:**

The inconsistency between HDSS and DHS approaches could have global implication on the estimation of child mortality and ethical issues on mortality inequalities. Further studies should be conducted to investigate the reasons of child mortality variation between the HDSS and the DHS approaches.

## Background

In the absence of complete vital registration system, low and middle-income countries (LMIC) use Demographic and Health Surveys (DHS) to monitor the health of their population [1, 2]. DHS collects nationally representative data including vital events using complex cross sectional cluster surveys at approximately 5-year intervals [3].

Over the last two decades, many countries across Africa and Asia have established Health and Demographic Surveillance System (HDSS) to monitor the demographic and health characteristics of a geographically defined population through continuous collection of vital events such as births, deaths and in-and out-migrations [1]. The HDSS provide a platform to conduct different studies and evaluate health programs and majority of them are affiliated to the INDEPTH Network [2].

There is some evidence to suggest that populations within an area covered by the HDSS have better health related outcomes such as health care seeking behavior compared to the populations not under continuous surveillance [4]. For example, maternal health care utilization such as antenatal care and health facility delivery were significantly higher in the Butajira HDSS compare to the communities outside of the Butajira HDSS [4].

Under-five mortality rate could be different in the HDSS sites compared to that of the DHS reports due to several reasons such as geographical variation (variations in risk factors in different geographic areas), methodological differences between DHS and HDSS and the study and/or surveillance effect in the HDSS sites. However, the under-five mortality rate discrepancy between the HDSS and DHS approaches has not been studied systematically. In this study, we investigated the trends of under-five mortality rates and annualized rate of mortality change between the HDSS sites and DHS reports in eight countries in Africa and Asia and 13 HDSS sites.

## Methods

### Settings

The study included 13 HDSS sites and 13 regions/districts in the same areas of the HDSS sites that had DHS reports in eight countries in Africa and Asia (Table 1). HDSS sites that were members of the INDEPTH network and had more than 2 years of surveillance data in the INDEPTH data repository [5] were included. HDSS sites that were not member of the INDEPTH Network or those who were members but did not have accessible data were excluded.

**Table 1:** HDSS sites and the corresponding DHS regions

Country	Name of the DSS	Location of the HDSS	HDSS data collection dates	Areas for the DHS reports	DHS data collection dates
Ethiopia	Gilgel Gibe	Ethiopia, Oromiya Region	2006–2011	Oromiya Region	2005, 2011
Kenya	Kisumu	Western Kenya, Nyanza province	2003–2008	Nyanza province	2003, 2008
	Kilifi	Coastal region of Kenya, Kilifi County	2003–2014	Coast province (both Kilifi and outside Kilifi)	2003, 2008, 2014
	Nairobi Urban	Nairobi slum area	2003–2014	Nairobi district including the city	2003, 2008, 2014
Uganda	Iganga	Easter Uganda, Iganga and Mayuge districts	2005–2011	Eastern region	2006, 2011
Tanzania	Magu	North-West Tanzania, Mwanza region, Magu district	1999–2010	Western Zone	1999, 2004, 2010
	Rufiji	Coastal region of Tanzania, eastern region, Rufiji district	2004–2010	Eastern Zone	1999, 2004, 2010
Malawi	Karonga	North Malawi, Karonga district	2004–2010	Karonga/Mzimba district	2000, 2004, 2010
South Africa	Agincourt	North-East South Africa, Mpumalanaga province	1998–2003	Mpumalanga province	1998, 2003
	Dinkale	South Africa, Limpopo Province	1998–2003	Limpopo/North province	1998, 2003
Senegal	Niakhar	West Senegal, Sine-Saloum Region	1993–2011	Fatick district, West Senegal	1993, 1997, 2005, 2011
	Mlomp	Senegal, Ziguinchor Region	1993–2012	Ziguinchor/southern region	1993, 1997, 2005, 2011
Vietnam	Filabavi	North Vietnam, Hanoi Region	2002–2011	Red River Delta region/rural area	1997, 2002, 2006, 2011

In the eight countries that are included in this study, DHS has been conducted approximately every 5 years using a nationally representative two-stage cluster sampling techniques. The DHS included several clusters (a cluster contains approximately 100 households) distributed by regions or districts in each country. For this study, we extracted the under-five mortality rates of the DHS reports in the same regions/districts of the HDSS sites in each country [6–25]. On the other hand, the HDSS collate longitudinal data in a geographically defined population in a region or district [1]. Even though both the DHS and HDSS sites were done in the same region/district, we couldn’t identify the overlapping villages in both approaches since DHS and HDSS did not report under-five mortality by villages. In addition to geographic variation, the time of data collection between the HDSS and DHS approaches could vary. To minimize bias related to time variation between the two approaches, we included DHS data that had less than two consecutive years gap with that of the HDSS.

### Data analysis

We used the microdata of the HDSS from the INDEPTH data repository [5]. Under-five mortality rates from the DHS reports in each region were extracted as stated above. Under-five mortality rates in the HDSS sites were determined using number of under-five deaths (numerator) and live births (denominator). The trends of under-five mortality rates using the HDSS and DHS approaches were compared by fitting exponential function using STATA 13. Annualized rate of change (ARC) of under-five mortality rates and 95 % CI were calculated for both the HDSS and DHS approaches.

## Results

**Fig. 1:**
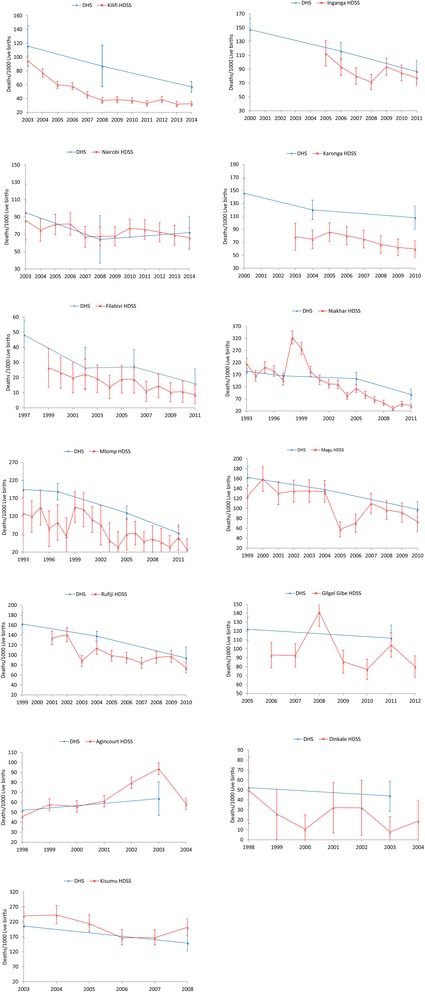
Trends of under-five mortality rates using DHS and HDSS approaches

The trends of under-five mortality rates in the HDSS are consistently lower than the under-five mortality rates of the DHS reports with the exception of the Kisumu and Agincourt HDSS sites in Kenya and South Africa respectively (Fig. 1).

In the Kilifi HDSS in Kenya, under-five mortality rate declined by 65.6 % between 2003 and 2014 with ARC of 12.2 % (95 % CI: 9.4–15.0). In the same period, the DHS under-five mortality rate in the Coastal region of Kenya declined by 50.8 % with ARC of 6 % (95 % CI: 2.0–9.0). The reduction of under-five mortality rate in the Nairobi HDSS (23.2 %) was comparable to under-five mortality rate decline in the DHS report in the Nairobi region (24.2 %) during 2003–2014. On the other hand, the Kisumu HDSS in Kenya had lower under-five mortality reduction (15.8 %) compared to the DHS under-five mortality decline (27.7 %) between 2003–2008 (Table 2).

**Table 2:** Annualized rate of change (ARC) of under-five mortality rates using DHS and HDSS approaches

HDSS site	Period	HDSS mortality change		DHS mortality	
		Median % change	ARC % (95 % CI)	Median % change	ARC % (95 % CI)
Kilifi	2003–2014	−65.6	12.2 (9.4,15.0)	−50.8	6.0 (2.0,9.0)
Nairobi Urban	2003–2014	−23.3	2.0 (0.7,2.5)	−24.2	4.0 (−42.0,35.0)
Kisumu	2003–2008	−15.8	6.4 (0.9,13.3)	−27.7	6.3 (5.6,6.6)
Karonga	2004–2010	−24.3	4.5 (1.5,7.3)	−10	3.0 (−6.0,12.0)
Rufiji	2004–2010	−34.6	5.0 (2.1,7.3)	−25.4	5.0 (−1,0,11.0)
Magu	1999–2010	−41.0	5.7 (2.4,9.0)	−39.6	4.5 (−1.8,11.0)
Iganga	2006–2011	−31.2	1.0 (−3.8,0.6)	−25	5.0 (−1.0,10.0)
Gilgel Gibe	2005–2011	−13.8	3.0 (−10.0, 14.0)	−8.2	1.7 (1.4,2.2)
Mlomp	1993–2012	−78.1	5.0 (2.2,0.8)	−62.5	4.4 (0,9.0)
Niakhar	1993–2011	−80.8	5.0 (0.8,9.0)	−52.2	3.4 (−3.0,10.5)
Agincourt	1998–2003	27.6	−7.2 (−20.9,4.5)	−18.4	4.1 (2.3,6.6)
Dinkale	1998–2003	−84	14.8 (−9.4,34.3)	−15.8	3.4 (3.0,4.8)
Filabavi	2002–2011	−56.9	8.4 (0.5,12.1)	−39.1	7.7 (−2.0, 16.0)

The under-five mortality rate in the Karonga HDSS in Malawi decreased by 24.3 % with ARC of 4.5 % (95 % CI: 1.5–7.3) between 2004 to 2010 while the corresponding DHS mortality rate declined by 10 % (ARC = 3, 95 % CI: 3.0–6.0). The Magu and Rufiji HDSS sites in Tanzania had higher under-five mortality reduction compared to the corresponding DHS under-five mortality rates. The under-five mortality rate reduction in the Mlomp (78.1 %) and Niakhar (80.8 %) HDSS sites in Senegal during 1993–2012 was significantly higher than the mortality decline observed in the DHS report during the same period.

In the Dinkale HDSS in South Africa, under-five mortality declined significantly compared to the DHS under-five mortality rates during 1998 to 2003. On the other hand, under-five mortality rate rose by 27 % in the Agincourt HDSS between 1998 to 2003 that was contrary to the 18 % under-five mortality reduction in the DHS report during the same period.

The Gilgel Gibe and the Filabavi HDSS sites in Ethiopia and Vietnam respectively had also higher under-five mortality reduction compared to the under-five mortality decline in the respective DHS reports.

## Discussion

Under-five mortality rates decline substantially in most of the sites during the last 10–15 years. Several factors, such as implementation of high-impact child survival interventions, health system strengthening, improvements in maternal education and family income, commitments of policy makers and donors and the declaration of Millennium Development Goals (MDGs) have contributed to a reduction in child mortality in LMIC [26].

Ten out of 13 (77 %) HDSS sites have consistently lower under-five mortality rates than that of the DHS reports. In the ten sites, the ARC and the median % of change of under-five mortality rates were higher in most of the HDSS sites than the DHS reports. However, in most of the sites (except Kilifi in Kenya), the 95 % CI of ARC in the HDSS sites and DHS reports overlapped (*P*-value > 0.05). The small sample sizes (number of years of observation) could resulted in non-significant findings. On the other hand, chance alone could not explain the huge differences in median % of change of under-five mortality rates between the HDSS and DHS approaches. Several other factors could explain the lower under-five mortality rates in the HDSS sites compare to the DHS reports. First, HDSS sites provide the platform for various interventions such as evaluation of immunization programs and the effect of insecticide-treated bed nets [2, 27, 28]. Such types of childhood interventions could have direct and indirect beneficial effects on child survival in the HDSS sites compared to the communities without the surveillance system. Second, the quality of maternal and child health care services within the HDSS sites is improved by the support of the interventions programs. For instance, coverage of health facility delivery in the Kilifi HDSS has increased from 15 % in 2005 to 28 % in 2013 (Deribew A, Moisi JC, Nokes DJ, Bauni E, Scott JA: Use of hospital-based morbidity surveillance to explain mortality trends in the Kilifi Health and Demographic Surveillance System, unpublished). Third, methodological differences between the HDSS and DHS could be a potential source of mortality variation between the two methods. DHS uses a two-stage cluster sampling methodology to collate information from women of reproductive age groups (residents and short term visitors) about history of births and deaths [29]. Sampling variation and recall bias could under-or overestimate child mortality rates in the DHS approach compared to the HDSS methodology. Use of long questionnaire by data collectors trained in short time may also introduce measurement bias in the DHS approach. On the other hand, HDSS collates mortality information longitudinally from a geographically defined population that may not be representative [1].

In some sites however, the HDSS mortality rates are higher or comparable to the DHS reports due to several reasons. The Kisumu and Agincourt HDSS sites have higher under-five mortality rates compared to the DHS under-five mortality rates. The high under-five mortality rate in Kisumu compared to the DHS under-five mortality rate could be explained by the high prevalence of malaria in the area. Some reports show that malaria is the leading cause of child mortality in Kisumu [30, 31]. The disruption of health services in Kisumu during the post-election period in 2008 would also have increased child mortality rates [30]. On the other hand, the Agincourt HDSS is located in one of the HIV prevalent areas in South Africa and has experienced mortality shock during 1990–2000 from HIV/AIDS causing an increase in child mortality rate than the DHS reports [32–34].

Under-five mortality rate in the Nairobi HDSS was comparable to the DHS report. The Nairobi HDSS is located in a densely populated urban slums with poor housing conditions and inadequate water and sanitation facilities which wipes out the survival advantages of urban settings [35]. In this instance, the DHS report that was conducted in Nairobi district could have lower under-five mortality rates than that of the HDSS in the slum areas.

The inconsistency between HDSS and DHS on mortality estimation has several global implications. In LMIC, child mortality is estimated almost exclusively by DHS and national census. The DHS methodology may estimate mortality rate differently compared to the HDSS. Utilization of DHS reports exclusively in LMIC could have overestimated mortality rate that has global implication on the accuracy of under-five deaths. The Global Burden of Diseases and Risk factors (GBD) [26] and the UN interagency group [36] have been mainly utilizing the DHS for mortality estimation and gave little attention for HDSS data. These groups and others have to consider several data sources including HDSS to accurately estimate mortality during the Sustainable Development Goals (SDG) era. Without accurate estimation of under-five mortality in each country, proper planning and resource allocation would not be possible. On the other hand, if the health interventions in the HDSS have positive impact on child survival, it will be unethical for the HDSS and other stakeholders not to address the high mortality rates in the communities outside of the surveillance system.

This study provides firsthand evidence on the variation of under-five mortality rate between the HDSS and DHS methodologies. However, the study has a number of limitations. DHS covers large geographic areas compared to HDSS and both approaches may not be conducted in the same years. Hence, the difference in under-five mortality rates between HDSS and DHS could be explained by time and geographic variations. However, geographic variation may not explain the low trends of child mortality in the HDSS compared to the DHS since most of the HDSS are located in areas of high mortality. We could not able to see the long-term mortality pattern between HDSS and DHS due to the small sample size (number of years of follow up).

## Conclusions

In conclusion, under-five mortality rates in the HDSS are generally lower than the DHS under-five mortality rates. The inconsistency between HDSS and DHS approaches could have global implication on the estimation of child mortality and ethical issues on mortality inequalities. Further studies using qualitative and quantitative approaches should be conducted to investigate the various reasons of child mortality difference between the HDSS and the DHS approaches.

## Abbreviations

ARC: Annualized rate of change
CI: Confidence intervals
DHS: Demographic Health Survey
HDSS: Health and Demographic Surveillance System
HIV/AIDS: Human immunodeficiency Virus/Acquired Immunodeficiency Syndrome
INDEPTH: International Network for the Demographic Evaluation of Population and Their Health
LMICS: Low and middle income countries
SDG: Sustainable Development Goals
